# KL2 mentored career development programs at clinical and translational science award hubs: Practices and outcomes

**DOI:** 10.1017/cts.2019.424

**Published:** 2019-12-26

**Authors:** Christine A. Sorkness, Linda Scholl, Alecia M. Fair, Jason G. Umans

**Affiliations:** 1 Institute for Clinical and Translational Research, University of Wisconsin-Madison, Madison, WI, USA; 2 Meharry-Vanderbilt Alliance, Nashville, TN, USA; 3 Georgetown-Howard Universities Center for Clinical Translational Science, Washington, DC, USA

**Keywords:** Mentored career development, clinical and translational science awards, clinical and translational research workforce, training curriculum, survey outcomes

## Abstract

**Introduction:**

NIH Clinical and Translational Science Awards (CTSAs) include KL2 mentored career development awards for faculty commencing clinical and translational research. A survey of KL2 leaders revealed program practices, curricular elements and compelling data about scholar characteristics and outcomes.

**Methods:**

We conducted a literature review, framed the survey construct, and obtained input from across the CTSA consortium. A REDCap survey was emailed in fall 2016 to 61 active programs.

**Results:**

Fifty-five programs (90.2%) responded. Respondents had been funded from 3 to 11 years, including 22 “mature” hubs funded for ≥8 years. Program cohort sizes were 56% “small”, 22% “medium”, and 22% “large.” Hubs offer extensive competency-aligned training opportunities relevant to clinical and translational research, including graduate degrees, mentorship, and grant-writing. Seventy-two percent of hubs report parallel “KL2-equivalent” career development programs. All hubs share their training and facilitate intermingling with other early stage investigators. A total of 1,517 KL2 scholars were funded. KL2 awardees are diverse in their disciplines, research projects, and representation; 54% are female and 12% self-identified as underrepresented in biomedical research. Eighty-seven percent of scholars have 2–3 mentors and are currently supported for 2–3 years. Seventy-eight percent of alumni remain at CTSA institutions in translational science. The most common form of NIH support following scholars’ KL2 award is an individual career development award.

**Conclusions:**

The KL2 is a unique career development award, shaped by competency-aligned training opportunities and interdisciplinary mentorship that inform translational research pathways. Tracking both traditional and novel outcomes of KL2 scholars is essential to capture their career trajectories and impact on health.

## Introduction

Clinical and Translational Science Awards (CTSAs), launched in 2006, create academic homes for clinical and translational research (CTR) and provide vital resources to develop the translational scientific workforce, including KL2 mentored research career development programs to support early stage translational investigators.

The initial CTSA requests for applications required programs to demonstrate how mentors and KL2 scholars would be trained and evaluated. NIH leadership recognized the importance of career development strategies by convening a CTSA Education Key Function Committee (KFC) “mentor working group”, which included the Research Education and Career Development Directors from 18 CTSA hubs. Their charge was to identify the types of initiatives developed by CTSA hubs to support KL2 scholars, which resulted in a series of “white papers” focused on approaches to strengthen the career development of clinical translational scientist trainees [1], and elements of mentoring [2–7]. Most reports of specific KL2 program practices and scholar outcomes focus on single institution experiences, representing large, unique, and/or well-resourced programs of greater longevity [8–11]. Given the evolution of CTR core competencies [12–13], of heterogeneous approaches to KL2 scholar training, and expansion of the CTSA consortium to include newer programs with smaller scholar cohorts, this survey now focuses on CTSA hub-reported program practices and scholar outcomes in the contemporary era.

## Methods

The survey was conducted under the aegis of the NCATS Workforce Development Domain Task Force which was charged to promote high-impact educational practices for scholars pursuing careers in CTR. A survey working group (WG) of this Domain Task Force was formed in May 2016 to design and implement a KL2 program leader survey of hub practices, as well as scholar characteristics and outcomes. An extensive literature review of career development programs and outcomes framed the survey construct. Input on survey content and administration was sought during 2017 CTSA Workforce Development Domain Task Force teleconferences and annual meetings. The survey expressly asked programs to report on their application and selection processes, scholar eligibility criteria, program size and directorship, duration of KL2 scholar support, parallel career development programs, training opportunities aligned to CTR competencies [12–13], and mentoring practices (“Career Sustainability Survey of CTSA KL2 Programs”, Supplementary Material). KL2 scholar characteristics and outcomes were reported by each hub in aggregate for all scholars appointed to date (i.e., not individual scholar-level data), and included data on applicant and scholar demographics, degrees at entry, primary appointments, certificates and degrees earned prior to and during the award period, transitions of KL2 scholars to individual career development awards and R01s, and scholars’ extramural funding patterns during and after the KL2, either as a principal- or co-investigator. The survey queried scholars’ career paths and research effort after KL2 program completion. Finally, respondents were asked to provide additional data about the survey completion process (i.e., unasked questions that they believed were important to evaluate the impact of their programs and the number of person-hours needed to complete this survey).

The survey was constructed in REDCap [14] and contained a combination of single- and multiple-response items, Likert scales, data table uploads, and short open-text responses. A penultimate survey draft was circulated to six KL2 program directors and their teams who beta-tested the draft and provided critical feedback that informed a revised final survey. Throughout the survey development process, the WG sought to balance gathering a rich set of program/scholar data against overburdening potential respondents, which might limit participation.

The survey was emailed in fall 2016 to the 61 active CTSA KL2 program PIs, with three subsequent reminders to encourage completion. The survey invitation to the PIs outlined the types of data that would be needed to answer the survey, as well as the assurance to share and publish only the aggregate data. Survey data were exported from REDCap to an Excel workbook for analysis. Descriptive analyses were performed on aggregate data from all responding CTSA hubs; a secondary analysis was performed on “mature” hubs, defined as operational for 8 years or longer, based on total duration of support, documented via NIH Research Portfolio Online Reporting Tools (RePORT) [15].

## Results

### Program Data

#### Respondent KL2 programs

Respondents from 55 KL2 programs (of 61 eligible CTSAs; 90.2%) completed the survey and provided usable data. The duration of CTSA funding ranged from 3 to 11 years; 22/55 (40%) were classified as “mature” hubs, as defined above. Current CTSA KL2 program sizes were reported as: Small (4 or fewer KL2 slots) 56%; Medium (5–6 slots) 22%; or Large (7–9 slots) 22%. The largest number of CTSA-funded KL2 slots *at any one time* in the program ranged from 2 to 27 (median 6). The *cumulative number* of CTSA-funded KL2 scholars by survey closure in summer 2017 was 1,517, ranging from 3 to 124 scholars per hub (median 20); 69% of these programs had appointed ≤30 scholars.

#### KL2 application process

**Fig. 1.**
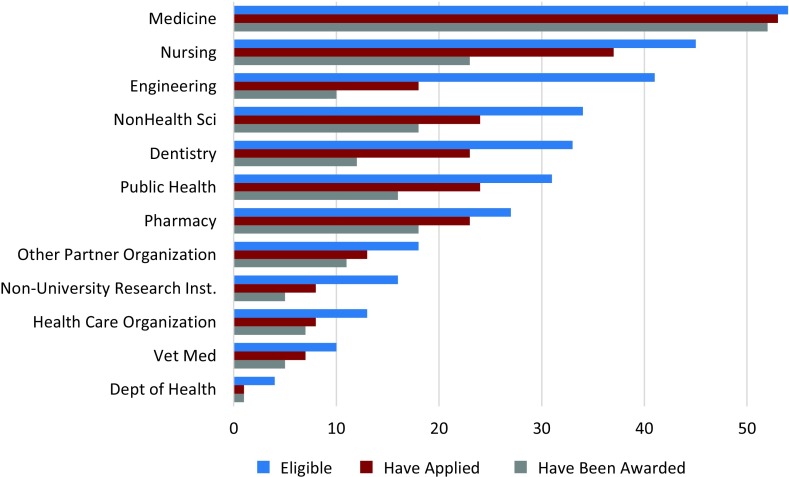
Number of Institutions with Eligibility, Applications, and Awards by Unit (*n* = 55). Bar chart showing the extent to which potential KL2 scholars from various units (e.g., medicine, nursing, engineering, etc.) across the 55 reporting hubs are eligible for, have applied for, and have been awarded KL2 scholarships.

The current KL2 application process at all hubs generally mimics the NIH individual K-series format and scholar selection criteria, that is, potential of the applicant to become a successful CTR investigator; quality of hypothesis-driven research proposal; feasibility in a 2 year time frame; mentor(s) and track record; training plan; research plan. CTSA hubs reporting current KL2 program partner eligibility, applications, and awards are summarized in Fig. 1. The largest proportion of eligible, applied, and awarded positions were housed in schools/colleges of medicine (98%, 96%, and 94.5%, respectively), with other academic units represented. The largest gap between applications and awards was in nursing; the largest gap between eligibility and applications was in engineering. CTSA hubs valued the following criteria in assembling a KL2 selection committee: representation of major CTSA partners/departments (59%), diversity of research focus across the translational spectrum (57%), and demographic diversity (28%). In addition, most programs (72%) utilized subject matter expert reviewers who were not selection committee members, with 62% of programs engaging external reviewers if internal expert reviewers were unavailable.

**Fig. 2.**
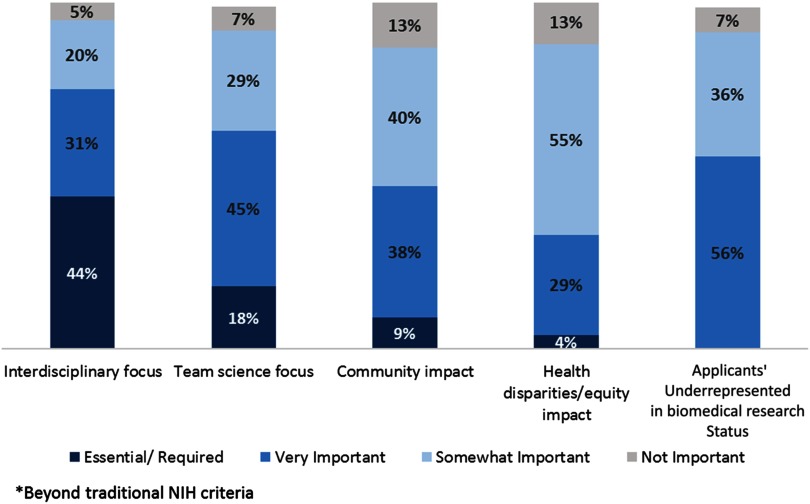
Project- and Applicant-Focused KL2 Selection Criteria. Bar chart showing the importance of various criteria beyond traditional National Institutes of Health criteria in determining which KL2 applicants and related research projects are selected for KL2 awards.

Scholar selection was based on both individual (scholar-level) and program (or cohort)-level criteria. At the scholar level, interdisciplinary and team science foci were cited as “essential/required” or “very important” (Fig. 2). At the cohort-level, demographic (69%) and disciplinary (64%) diversity were ranked most highly. The percent of KL2 applicants typically funded ranged from 2 to 70% (median 18%); 36 programs (66%) reported selection of <20% of their applicants.

#### KL2 scholar eligibility

The percentage of hubs reporting scholar academic ranks as “KL2-eligible” were: 98% assistant professors; 83% instructors; 20% associate professors; and 22% others (pre-academic rank, researcher/scientist, research associate, fellow, clinical resident). Eligible tracks for a KL2 award reported by programs were: 95% tenure seeking; 95% research; 75% clinical; and 9% others.

#### KL2 program directorship

Current KL2 directors were reported as academically seasoned, primarily physicians, and from senior academic ranks. Their degrees included: 48% MD only; 22% MD/PhD; 15% PhD only; 9% MD/MPH; and 6% other (PhD/DDS, PharmD only, PhD/PharmD). Five percent of KL2 directors had additional education degrees. 15% of directors had K24 mid-career support. KL2 leadership was stable over the life of the program, as reported by 87% of the hubs. 25% of programs reported one primary director who mentors KL2 co-directors for leadership sustainability; 55% reported co-directors with complementary skills and responsibilities to the contact KL2 PI.

#### KL2 scholar support

**Fig. 3.**
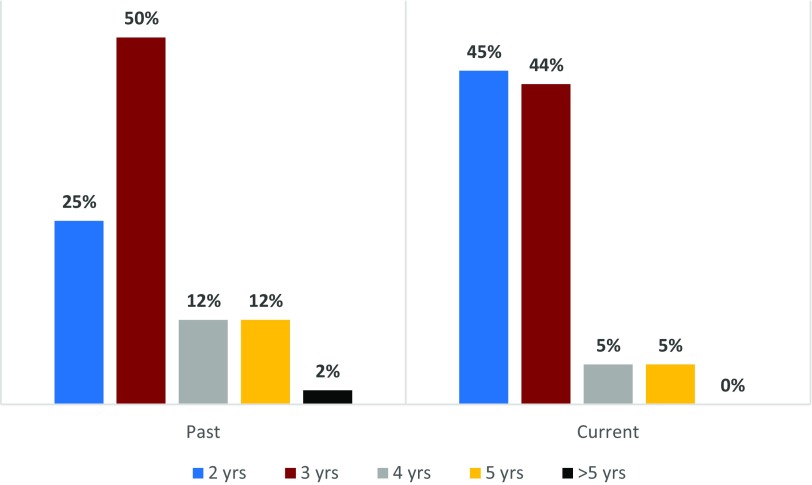
Maximum Number of Years of CTSA-Support for KL2 Scholars (Past and Current). Bar chart showing the percent of hubs reporting that their KL2 scholars receive 2, 3, 4, 5, and 5+ years of KL2 support from the Clinical and Translational Science Award program in the past and currently.

The hub-reported maximum duration of KL2-funded support, both past and present, is shown in Fig. 3. The majority of scholars were supported for 2–3 years, 75% in the past and 89% at present, with a recent shift from 3 years or longer to only 2 years of support. The percentage of scholars supported for >3 years decreased from 26% for past scholars to 10% for present scholars. The majority (55%) of hubs *now* use institutional funds to *extend* the duration of scholar support; 30% provided support *after* the KL2, 25% supported *prior* to the KL2.

#### Parallel career development programs

Parallel internal or extramurally-funded “KL2-equivalent” career development programs were reported by 72% of hubs. Total slots per hub in these parallel programs ranged from 1 to 36 (median 10). Funding sources included: NIH K12 (45%), institutionally-funded/internal (63%), extramural/non-federal extramural (18%), and other (7%). All CTSA hubs with such parallel career development programs reported sharing their KL2 training opportunities, such as seminars/workshops and graduate courses with these scholars.

#### KL2 program training opportunities

KL2 programs offered extensive training in: grant writing (96%), team science (89%), mentorship (82%), scientific writing (75%), entrepreneurship (69%), leadership (65%), as well as off-site experiences (53%), “research in progress” seminars (87%), and others (33%). Fifteen programs reported engagement in off-site experiences, for example, externships or mini-sabbaticals, with a median of 4 (range 1–22) scholars engaged. Other training topics and programs included: good clinical practice, responsible conduct of research/ethics, mentee training, community-engaged research, cultural competency, health disparities research, project management, research administration, and communication. 87% of KL2 programs reported that scholars with individual K awards (or equivalent) could access these KL2 offerings.

#### Contributions of KL2 graduates to the CTSA program

When asked how KL2 program graduates contributed to their institution’s CTSA program, most common roles were: acting as mentors (84%), reviewing pilot award applications (73%), and facilitating K writing groups (26%). Less common contributions included: serving on K or T applicant review panels, participating in KL2 seminars, serving on KL2 advisory groups, and teaching.

#### KL2 program mentoring practices

The programs were queried as to the *current* minimum size of the KL2 mentor team. Results, in order of frequency, were: 3 mentors (47%), 2 mentors (40%), 0–1 mentors (9%), and 4–5 mentors (4%). Only 7% of programs reported that mentors were “compensated in any way”; compensation methods included fractional salary coverage, mentor stipends, and non-salary funds to support the mentoring relationship. Formal Individual Development Plans (IDPs) are *currently* required of scholars by 89% of KL2 programs. Whereas the majority of KL2 programs *offered* mentor training, only 25% currently *require* it, with a median minimum requirement of 4 (range 1.5–16) hours. The frequency of scholar meetings with their program mentors and KL2 leadership varied widely. The majority of scholars met regularly with their entire mentoring teams quarterly (30%), biannually (32%), or annually (10%). The scholar/mentor teams also had regular meetings with KL2 leadership, reported as quarterly (30%), biannually (32%), and annually (10%). Scholars met with *any* member of their mentoring teams more frequently, reported as weekly (61%), semi-monthly (25%), and monthly (12%).

### Scholar Data

#### Scholar demographics

Females were represented similarly among applicants (median 51%, range 33%–80%) and awardees (median 54%, range 13%–83%). Likewise, the proportion of individuals who self-identify as underrepresented in biomedical research (URBR) was similar among applicants (median 15%, range 0%–31%) and awardees (median 12%, range 0%–25%). Of note, 8 CTSA hubs contributed >25% of total URBR scholars.

#### Scholar primary academic appointments at KL2 program entry

Most hubs (87%) reported that >50% of their KL2 scholars entered with an “Academic Faculty” primary appointment; 38% reported that all KL2 scholars entered with that title classification. Other types of primary appointment titles were reported less commonly. For example, 24 hubs (46%) reported scholar entry as “Lecturers”/ “Instructors”/ “pre-faculty title”; however, those titles and ranks generally represented <25% of their overall scholar population. A similar pattern was noted with the title “Scientist/Researcher”. Only four hubs reported entry of any KL2 scholars with a “Hospital” title.

#### Scholar academic degrees at KL2 program entry

Survey participants estimated the percent of scholars to date who had entered with clinical degrees only (e.g., MD, DVM, DDS, PharmD), with research degrees only (non-clinical PhDs), and with both clinical and research degrees. Across all reporting hubs, it was more common for scholars to enter with clinical degrees only (median 40%; range 0%–90%). Hubs reported that a median of 27% entered with a research degree only (range 0%–60%) and a median of 25% (range 0%–95%) entered with both clinical and research degrees.

#### Scholar training in clinical and translational research at entry and during the KL2

Hubs estimated percentages of their scholars who had completed a certificate or degree in CTR prior to their KL2 training. Most hubs (80%) indicated that 25% or fewer of their scholars had done so. Overall, the median reported percent of scholars who had such degrees/certificates was 9%. Interestingly, four hubs reported that 50%–95% of their KL2 scholars entered with a degree or certificate in CTR. By contrast, scholars completed significant didactic training during the KL2 including a median of 20% (range 0%–100%) enrolled in certificate and/or degree programs and 80% (range 0%–100%) in individual courses, likely influenced by prior didactic and research experiences and guided by their IDPs.

### Scholar Outcomes

#### Transitions to individual career development awards and R01s

**Fig. 4.**
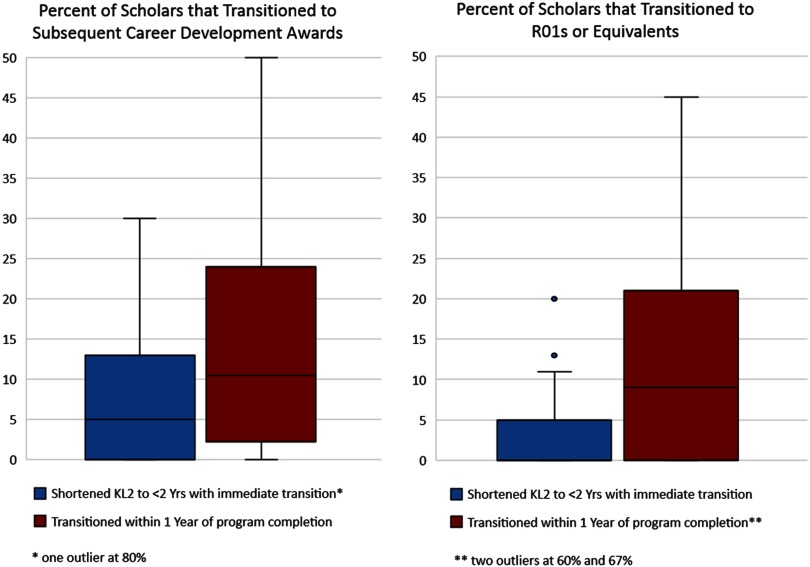
Transitions to Career Development and R01 Awards (hub reported). Box chart indicating the percent of KL2 scholars that transitioned immediately and within 1 year of program completion to subsequent career development awards and R01s or equivalents.

The majority of hubs reported that <40% of their KL2 scholars shortened their CTSA support to <2 years by transitioning to individual K- or R01 or equivalent grant support. However, one hub reported that 80% of their scholars transitioned early. By contrast, as shown in Fig. 4, a larger proportion of scholars transitioned to either an individual career development award or to an R01 or equivalent grant within 1 year following completion of CTSA support.

#### CTSA scholars’ career paths and research effort after KL2 program completion

Hubs were asked to report KL2 scholar career paths and to estimate research effort of their graduates within 3 years of program completion and beyond; 47/55 responding hubs (85%) provided such data. The majority of KL2 graduates were retained by the CTSA hub at which they trained (78% at 3 years and 63% beyond 3 years); others moved to a different academic institution (17% at 3 years and 24% beyond 3 years). The remainder reported non-academic employment by government or non-profit organizations, industry, the private sector, or hospitals.

**Fig. 5.**
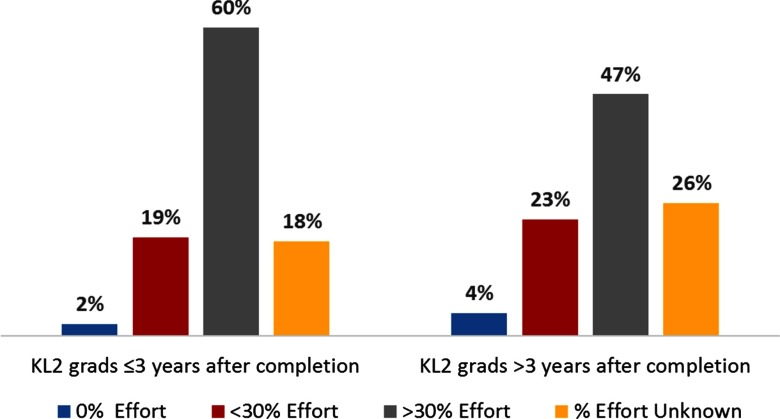
KL2 Graduates’ Percent Research Effort since Program Completion. Bar chart indicating the percent of scholars who have 0%, <30%, >30% research effort or % effort unknown, both ≤3 years and >3 years following program completion.

Figure 5 describes retention of CTSA KL2 graduates in CTR efforts, as estimated by the reporting hubs. Within the first 3 years of KL2 completion, 60% of alumni devoted >30% effort and 21% devoted < 30% to CTR. Beyond 3 years of KL2 completion, alumni’s CTR effort was estimated to decrease (47% devoted >30% effort and 27% devoted <30% to CTR).

#### Extramural research funding of CTSA scholars during the KL2 program and beyond

**Fig. 6.**
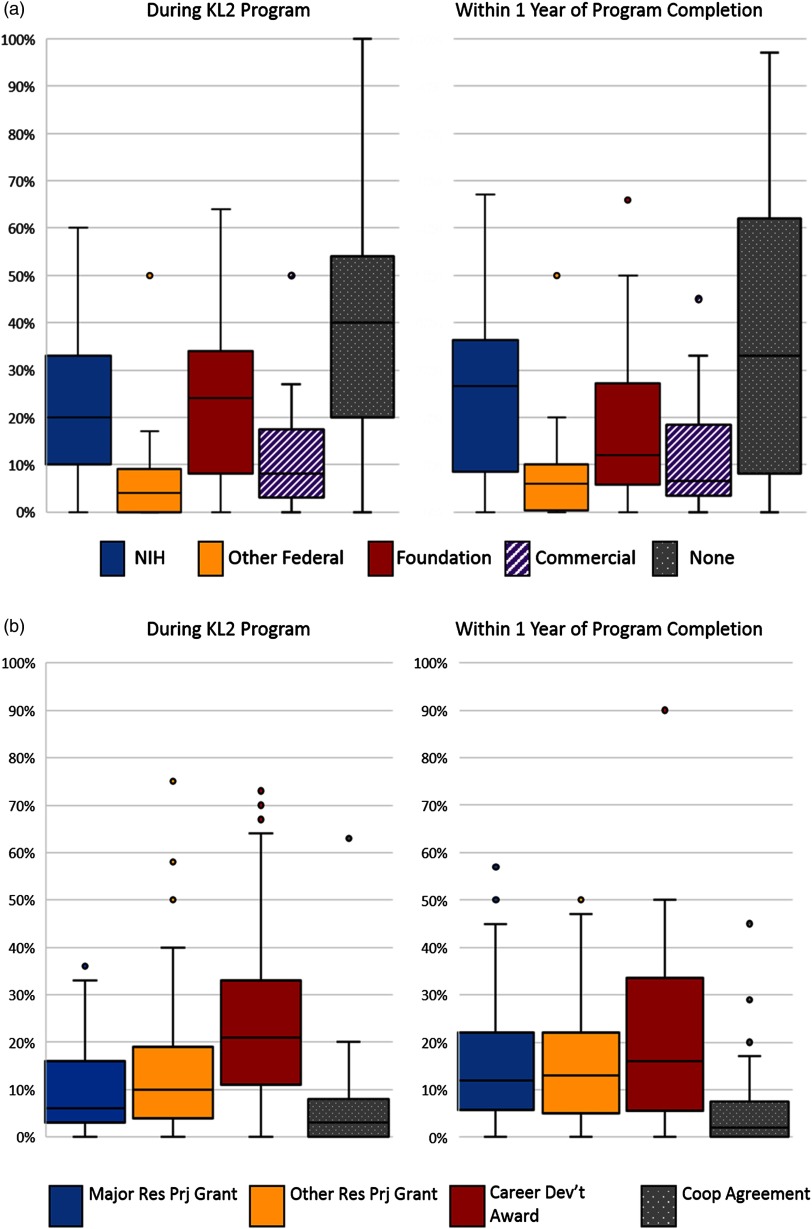
Percent of Scholars Awarded Extramural Funding as PI During and After KL2 Program by Funding Source (hub reported). **(a)** Box chart indicating the percent of scholars as reported by hubs who received extramural funding as a PI, both during and after program completion by the source of funding. Scholars received additional support during the KL2 from the National Institutes of Health (median of 20%); other federal, for example, Veteran’s Administration, Agency for Healthcare Research and Quality, National Science Foundation (median of 4%); foundation, for example, American Heart Association, American Cancer Society, Robert Wood Johnson, Doris Duke (median of 24%); and commercial (median of 8%). Scholars received support within 1 year of KL2 from the National Institutes of Health (median of 27%), other federal (median of 6%), foundation (median of 12%), and commercial (median of 7%). **(b)** *Percent of Scholars Awarded Extramural Funding as PI During and After KL2 Program by Funding Mechanism (hub reported)*. Box chart indicating the percent of scholars as reported by hubs who received extramural funding as a PI, both during and after program completion by the mechanism of funding. Scholars received additional support during the KL2 through a major research project grant, for example, R01, Program Project Grant, Veterans Administration Merit, or equivalent (median of 6%); other research project grant, for example, R03, R21, or equivalent (median of 10%); career development award, for example, federal or foundation (median of 21%), and cooperative agreement and contracts (median of 3%). Scholars received support *within* 1 year of KL2 through a major research project grant (median of 12%), other research project grant (median of 13%), career development award (median of 16%), and cooperative agreement and contracts (median of 2%).

Figure 6a and b outline the sources (NIH; other federal, e.g., VA, AHRQ, CDC, NSF; foundation/professional society, e.g., AHA, ACS, RWJ, Doris Duke; or commercial) and mechanisms (major research project grant, e.g., R01, program or center grant projects, VA Merit or equivalent); other RPG (e.g., R03, R21 or equivalent; individual career development award; or cooperative agreement/contract) of extramural funding to CTSA scholars as a principal investigator (PI) during the KL2 program, and within 1 year of completion. As expected, these funding sources were diverse. *During the period of KL2 support*, the 55 responding hubs reported that their scholars received additional support primarily from NIH and foundation sources. We note that this response represents a composite of additional research support that might enhance KL2 scholar productivity by running concurrently with the KL2 award plus new awards that would support direct transition (i.e., without a funding hiatus) at the end of the KL2 program. A career development award was the most common funding mechanism. *Within 1 year of KL2 program completion*, NIH remained the most common source of additional support; career development awards were the most common funding mechanisms, followed closely by RPGs (both major and other).

**Fig. 7.**
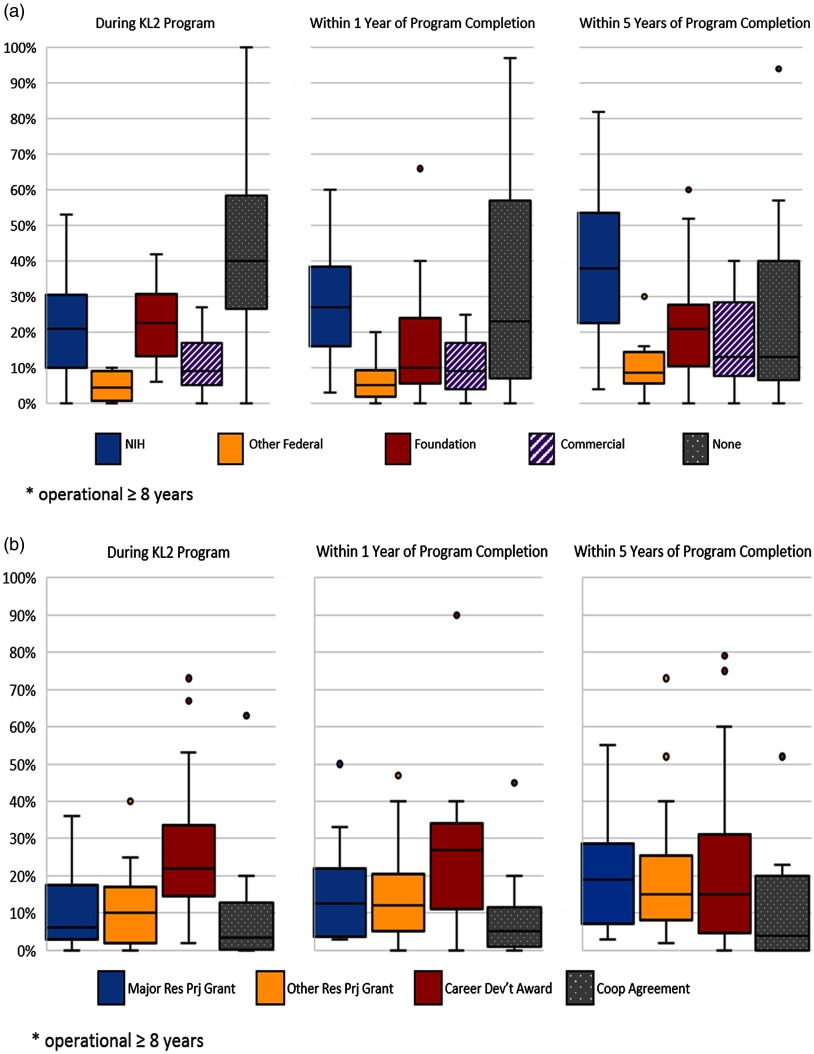
Percent of Scholars Awarded Extramural Funding as PI During and After KL2 Program by Funding Source (at Mature Hubs). **(a)** Box chart showing the percent of scholars as reported by mature hubs (i.e., operational for ≥8 years) who received extramural funding as Principal Investigator, both during and after program completion by the source of funding. Scholars received additional support *during the KL2* from the National Institutes of Health (median of 21%); other federal, for example, Veterans Administration, Agency for Healthcare Research and Quality, National Science Foundation (median of 5%); foundation, for example, American Heart Association, American Cancer Society, Robert Wood Johnson, Doris Duke (median of 23%); and commercial (median of 9%). Scholars received support *within 1 year of KL2 completion* from the National Institutes of Health (median of 27%), other federal (median of 5%), foundation (median of 10%), and commercial (median of 9%). *Within 5 years of program completion*, scholars received support from the National Institutes of Health (median of 38%), other federal (median of 9%), foundation (median of 21%), and commercial (median of 13%). **(b)** *Percent of Scholars Awarded Extramural Funding as PI During and After KL2 Program by Funding Mechanism (at Mature Hubs)*. Box chart indicating the percent of scholars as reported by mature hubs (i.e., operational for ≥8 years) who received extramural funding as Principal Investigator, both during and after program completion by the mechanism of funding. Scholars received additional support *during the KL2* through a major research project grant, for example, R01, Program Project Grant, Veterans Administration Merit, or equivalent (median of 6%), other research project grant, for example, R03, R21, or equivalent (median of 10%), career development award, for example, federal or foundation (median of 22%), and cooperative agreement and contracts (median of 4%). Scholars received support *within 1 year of KL2 completion* through a major research project grant (median of 13%), other research project grant (median of 12%), career development award (median of 27%), and cooperative agreement (median of 5%). *Within 5 years of program completion*, scholars received support through a major research project grant (median of 19%) other research project grant (median of 15%), career development award (median of 15%), and cooperative agreement (median of 4%).

Figures 7a and b summarize the reported extramural funding sources and mechanisms from the subset of “mature” hubs (*n* = 22), as these were the only ones able to provide data for longer-term outcomes following KL2 completion. Award sources and grant mechanisms during and within 1 year after KL2 support were similar in this subset to those reported by the entire cohort. Within 5 years of program completion, NIH was the most common funding source for KL2 alumni, and major RPGs were the most common funding mechanism.

The survey instrument queried this same information for *co-investigator* extramural funding sources and mechanisms. However, data were insufficient in quantity and/or quality to conduct an analysis for either the entire hub cohort or the “mature” sample, with most hubs reporting that they lacked this information.

#### Respondent suggestions for additional data collection of importance

In the final section of the survey, respondents were asked to list additional types of data related to KL2 scholar outcomes that they considered important, but not requested. Responses included: (a) availability of pre-K pipeline programs; (b) grant-related outcomes (e.g., year-by-year rates of submission, including those unfunded; age when awarded first R01; time from KL2 to first R award (of any type); and return on investment); (c) publications (e.g., number before, during, and after award; number that were first-authored; (d) indicators of research impact; (e) other outcomes such as patents and inventions; and (f) career trajectory (e.g., promotions, tenure, and leadership roles).

## Discussion

The success of a sustainable and diversified biomedical research workforce depends on recruiting and retaining talented investigators, mentoring them to career independence, and ensuring their persistence and progression in translational research. This is the first CTSA-wide KL2 survey to gather information from CTSA hub leaders to identify key program practices and curricular elements that may contribute to success, as well as data about their scholar characteristics and outcomes. However, we note the hybrid nature of our survey data, as they reflect contemporary training practices but outcomes for scholars whose past training experiences may have differed.

Despite the 90.2% response rate, we acknowledge the inherent limitations of hub-reported aggregate data that rely on retrospective recall coupled with the use of program files and records, public databases, and data used to complete annual Research Program Progress Reports (RPPRs). Importantly, these data are likely representative of the KL2 programs nationally and more generalizable than—but complementary to—single CTSA hub KL2 program reports [8–11] or reports from regional consortia [16]. Data from this survey also complement results reported by Guerrero *et al*. [17], based on an analysis of 2006–2013 data and focused on institutional characteristics associated with large numbers of K awards.

The NIH has identified the need to bridge the gap between basic, clinical, and population research by supporting the career development of clinical and translational scholars [18–20]. Our survey results document the ability of CTSA programs to successfully attract and train scholars on this career path. True to the CTSA mission, the KL2 cohorts are diverse in multiple ways in accord with the goals of CTR and emphasizing interdisciplinary collaboration and team science.

These data also confirm that KL2 program eligibility criteria, scholar application and selection processes, and scholar and cohort selection criteria create an interdisciplinary and team science learning community. KL2 programs inclusively invite and accept scholars from multiple disciplines and academic affiliates, and influence creation of diverse scholar cohorts to achieve health impact. Programs also consider “community impact” and “health disparities impact” of applicants and their research projects among selection criteria. While achieving apparent gender balance among awardees, KL2 programs have not accomplished full inclusion of underrepresented groups. Enhancing this applicant pipeline will be essential to achieving diversity in our KL2 programs. In addition, program directors reported that representation of CTSA hub partners/departments, as well as diversity of demographics and research focus across the translational spectrum, influence the intentional creation of KL2 cohorts and learning communities. These data indicate KL2 program alignment with CTSA goals and the evolution of a framework to inform personalized training pathways for CTR phenotypes.

Survey data highlight the KL2 program elements and practices that may be influential and attractive to early stage investigators (ESIs). KL2 PIs are generally senior investigators who engage and mentor complementary co-leadership for program stability and sustainability. The extent of outstanding and diverse training opportunities is impressive and unique, aligned with the evolving CTR competencies [12–13]. The KL2 training portfolio includes: traditional grant and scientific writing; contemporary offerings of team science, entrepreneurship, leadership, community engagement, and health disparities research; evidence-based research mentor and mentee training [21]; and utilization of IDPs. Trainings address the organizational factors that are critical for development of successful clinical and translational scientists [22], as well as critical career development program components that go beyond didactic training [23]. For those without prior research degrees, many complete CTR certificates or degrees; individual coursework is widely completed by others.

There are two major threats to the growth in numbers of the KL2 scholar phenotype. First, limited funding and decreased training slots make most KL2 awards highly competitive; two-thirds of hubs report funding <20% of applicants. Along with the decreased duration of KL2 support (now most commonly 2 years), this may limit both the impact and reach of KL2 programs. In response to this shortened support and limited cohort size, a majority of hubs are implementing approaches to extend the duration of protected time for these ESIs to gain sufficient training and research experience. Options include: (1) extending scholar support beyond the 2 years of KL2 support via institutional mechanisms (55% of hubs); (2) advising scholars to submit applications for individual K awards early in their KL2 for up to 5 additional years of support; or (3) advising ESIs to preferentially apply for an independent K (rather than to their own KL2 programs) due to the immediate assurance of longer support, coupled with more favorable published award rates. An additional, predictable consequence of shortened duration of KL2 support is to target submission of an individual K award or RPG application as early as the first year to avoid an early funding hiatus. Were it not for institutional support to extend the duration of protected KL2 training, scholars would be caught between the dual threats of decreased retention in CTR due to loss of funding and inadequate competency-aligned training during the period of mentored career development.

The highly competitive selection of KL2 scholars contrasts with the higher success rates for most individual K-series awards. NIH RePORT 2018 data indicate average success rates for K01 (31.0%), K08 (39.7%), and K23 (37.7%) applications, that vary by numbers of applications and individual institutes [15]. The analysis published by Guerrero *et al*. [17] demonstrated that CTSA institutions from 2006 to 2013 (*n* = 61) received a total of 4783 new independent K awards (most commonly K23, K08, or K01 mechanisms). Thus, the CTSA hub institutions and their ESIs have been successful in seeking these awards, both for primary support, as well as for extended support subsequent to a KL2 award. Second, recent reductions in KL2 program support and cohort size are concerning as they may result in loss of a “critical mass” of scholars who are trained according to a competency-aligned CTR curriculum with a discipline-spanning focus. Most of our scholar outcome data derive from larger, “mature” hubs and reflect their training durations and practices years ago. By contrast, most *current* scholars train in smaller and newer programs, for shorter durations, and with newer competencies and program practices. Therefore, we should be cautious in ascribing our scholar outcomes to current training practices.

Survey data provide strong evidence of KL2 program institutional impact beyond benefit to scholars themselves. First, the majority of KL2 programs share their curricula and broad training offerings including research mentor and mentee trainings, with other early career faculty. Second, KL2 scholars and alumni share their translational perspectives and skills within their home departments and research settings.

Metrics for success of CTSA career development awards include alumni employment and persistence in CTR careers [16], regardless of institutional setting. Survey results demonstrate continued research engagement and retention of alumni in the translational science workforce, primarily in academics.

KL2 programmatic success is also judged by grant awards and their timing. Hub-reported data on extramural funding sources and mechanisms highlight diversified research portfolios, as expected in this funding climate, which evolve longitudinally. NIH funding success rates are variable across the hubs, but impressive. The most common mechanism is the individual career development award, followed by an NIH RPG. These outcomes primarily reflect the subset of “mature” CTSA hubs, which tend to be larger and better resourced, and report on scholar outcomes that may no longer reflect current training. A striking gap in our data revealed the inability of hub leadership to track alumni funding in other than PI roles. Given the programmatic focus on collaborative team science, our inability to capture effort of KL2 alumni as funded co-investigators, a desired outcome for our graduates, reveals a near-universal weakness in scholar tracking.

It is important to compare KL2 program-reported outcomes on grant trajectory with comparable data from other relevant sources. At the April 2018 Translational Sciences meeting, NCATS Program Officer Joan Nagel, MD, MPH, presented an historical assessment of the CTSA Program KL2 Scholar Program (2006–2014) [24] using the NIH Grants Database: Query, View, Report (QVR), a data mining system that extracted data from eRA Commons and IMPAC II (accessed in August 2016). Dr. Nagel reported that 49% of all KL2 scholars (*n* = 952) from all degrees (2006–2014) went on to receive subsequent grants from NIH/Center for Disease Control (CDC)/Food and Drug Administration (FDA)/Agency for Healthcare Research and Quality (AHRQ) and the Veteran’s Administration (VA); PhDs received NIH grants exclusively. For MDs and MD-PhD scholars, their first subsequent grants were primarily individual K awards. By contrast, PhD scholars progressed primarily to R-series grants. Subsequent K- and R-series awards were supported by a wide range of NIH institutes and centers. These data, obtained from administrative NIH databases, are consistent with our survey evidence of subsequent KL2 scholar research and career development support, which additionally identified important non-federal sources. Importantly, these administrative data share the same weakness as our survey data by including only awards received as PI, failing to capture the full extent of research funding, research effort allocation, or collaborative science focus.

Evolving CTR competencies [12–13] and scholar needs will expand the growth of KL2 training components in contemporary skills such as clinical and health informatics, “big data science”, and dissemination and implementation science. Such offerings will likely be shared with other career development awardees and ESIs on hub campuses. This “intermingling” of KL2 and individual K scholars may influence research pathways and career choices, plus facilitate collaborative research partnerships and innovative grant applications to address human health. This sharing of KL2 training resources and the growing number of our alumni retained in academic health centers may contribute to shifting the research and training culture of junior faculty who are not supported by the KL2 mechanism toward discipline-spanning translational team science. By contrast with this speculative optimism, the shortened duration of KL2-support may compromise the rich competency-aligned training that CTSA programs pioneered due to the competing needs for mentored research training and productivity and the didactic and experiential activities to achieve core competencies. Coupled with the increasing transition of our graduates, especially clinician-scientists, to longer periods of mentored research career development supported by individual K-series awards (e.g., 2 years of KL2 support followed by either an individual K23 or K08 award), the specific impact of the KL2 training and pathway may be diluted or lost during longer and discipline-focused subsequent training. Unfortunately, we will not be able to determine which of these outcomes will predominate without longitudinal follow up of current KL2 trainees, including those who transition to individual K awards on their paths to independence.

By contrast with individual K-series awards, the KL2 is neither disease- nor discipline-specific, but rather is designed to provide career development of CTR investigators. KL2 training is shaped by competencies and interdisciplinary mentorship that inform this career pathway. CTSA hubs lead this novel and still-evolving training effort. It is our responsibility to track outcomes beyond publications, grants, and patents, as well as the unique career trajectories of KL2 alumni.

Will the CTSA KL2 Program serve as a true and unique career development program OR simply a method for “bridge funding” due to the threats described above? The ultimate impact of the KL2 program will require analysis of long–term outcomes of scholars completing the KL2 alone, the KL2 plus individual K award hybrid, and the individual K alone. We recommend that structured alumni surveys, including key impact metrics (ideally with a qualitative component to address career satisfaction and persistence) be conducted every 5 years, with concurrent KL2 program surveys. Such a cycled approach would provide complementary data and accommodate linking of scholar outcomes to program sizes, structures, and practices and to enable analysis of scholar subgroups. In addition, data from public sources such as NIH RePORT and NIH Grants Databases should be queried to reflect the same 5-year reporting periods. We do not trivialize the effort required to coordinate and gather systematic data across hubs, but raise the call for action.

## Supplementary material

For supplementary material accompanying this paper visit http://dx.doi.org/10.1017/cts.2019.424. click here to view supplementary material
