# Supplementary material for: KL2 mentored career development programs at clinical and translational science award hubs: Practices and outcomes
Source: J Clin Transl Sci. 2019 Dec 26;4(1):43–52. doi: 10.1017/cts.2019.424 (PMC7103475; doi:10.1017/cts.2019.424)
Supplement: Supplementary file 1 [file S2059866119004242sup001.pdf]

CTSA Clinical & Translational® Science Awards

Career Sustainability Survey Of KL2 Programs

Please complete the survey below. We estimate this survey will take xx minutes to complete.

Thank you!

INSTITUTIONAL FACTORS: Please indicate the extent to which the following schools/colleges/other CTSA partners currently participate in your CTSA Workforce Development KL2? (we realize that these participating units may have changed over time; please check all that apply now)

	Individuals from this unit are eligible to apply for a KL2 program	Individuals from this unit have applied for a KL2 award	Individuals from this unit have received a KL2 award	N/A
School of Medicine	☐	☐	☐	☐
School of Dentistry	☐	☐	☐	☐
School of Engineering	☐	☐	☐	☐
School of Nursing	☐	☐	☐	☐
School of Pharmacy	☐	☐	☐	☐
School of Veterinary Medicine	☐	☐	☐	☐
School of Public Health	☐	☐	☐	☐
Non-Health Sciences College (ex. Science (biology/chemistry), Education, Social Science)	☐	☐	☐	☐
Department of Health	☐	☐	☐	☐
Non-University Research Institute	☐	☐	☐	☐
Health Care Organization	☐	☐	☐	☐
Other Partner Organizations	☐	☐	☐	☐

If other please specify ____________________

APPLICATION AND SELECTION FACTORS

Does your application generally mimic the NIH K format and selection criteria (i.e., potential of the applicant to become a successful clinical/translational investigator; quality of hypothesis driven research proposal; feasibility in a two year time frame; mentor(s) and track records, training plan; research plan)?

○ Yes
○ No

Please indicate how important the following additional criteria are in your current review process when making individual KL2 awards.

	Essential/Required	Very Important	Somewhat Important	Not Important
Interdisciplinary focus of proposed research and training	☐	☐	☐	☐

Team science focus of proposed research	☐	☐	☐	☐
Community impact of proposed research	☐	☐	☐	☐
Health disparities/equity impact of proposed research	☐	☐	☐	☐
Applicant's membership in a group under-represented in the biomedical workforce (race, ethnicity, disability)	☐	☐	☐	☐

Please indicate how important the following criteria are in your current review process when putting together the KL2 cohort

	Essential/Required	Very Important	Somewhat Important	Not Important
Maximizing the diversity of the cohort with respect to research translational stage	☐	☐	☐	☐
Maximizing the diversity of the cohort with respect to demographic factors (eg. gender, race, ethnicity)	☐	☐	☐	☐
Maximizing the diversity of the cohort with respect to discipline	☐	☐	☐	☐

Please list any other additional criteria you use in your selection of KL2 scholars and/or the KL2 cohort.

Please indicate how important the following factors are in the makeup of your curent CTSA KL2 selection committee.

	Critical/Required	Very Important	Somewhat Important	Not Important
Committee has good representation from your major CTSA partners and departments	☐	☐	☐	☐
Committee reflects research foci across the translational spectrum	☐	☐	☐	☐
Diversity of the committee (i.e. gender, race, ethnicity)	☐	☐	☐	☐

Do you use specific subject matter expert reviewers who are not members of the selection committee?

- ○ Yes
- ○ No

Roughly what percent of your usual applicant pool do you fund? (ie. payline)

Other Eligibility Criteria

Applicants with the following faculty ranks (or equivalent) are eligible for a KL2 award (Check all that apply)

- ☐ Instructor
- ☐ Assistant Professor
- ☐ Associate Professor
- ☐ Other applicant eligibility (please specify)

Applicants on the following tracks are eligible for a KL2

- ☐ Tenure-seeking

award (Check all that apply)

- [ ] Research
- [ ] Clinical
- [ ] Other applicant eligibility (please specify)

CTSA and KL2 Program Factors

What is the current **funding level size and allowed number of KL2 slots of your CTSA program?**

- ( ) Small
- ( ) Medium
- ( ) Large

Small CTSA funding level size = less than or equal to 4, Medium CTSA funding level size=6, Large CTSA funding level size=9

Is your KL2 program currently **operating under these CTSA funding level sizes?**

- ( ) Yes
- ( ) No

What is the largest number of total CTSA-funded or authorized slots that your KL2 program has had at any one time?

Did you use institutional co-funding or cost sharing in order to fill that maximal number of slots?

- ( ) Yes
- ( ) No

What is the cumulative number of CTSA funded KL2-scholars appointed over the lifetime of your KL2 program?

Of the following scenarios, please check all that describe your K directorship.

- [ ] Little turnover of the senior directors over the life of the program
- [ ] Extensive turnover over the life of the program
- [ ] One primary director who currently mentors co-directors for sustainability
- [ ] Current co-directors with complementary skills and responsibilities to those of the main/contact KL2 PI

Current KL2 Program PI/Director (main/contact PI) Degree (check all that apply)

- [ ] MD
- [ ] PhD
- [ ] Other

Does the current KL2 Program PI/Director (main/contact PI) have an education related degree?

- ( ) Yes
- ( ) No

What other relevant training does your KL2 Program PI/Director (main/contact PI) have?

Has the current KL2 Program PI/ Director (main/contact PI) ever been K24-supported?

- ( ) Yes
- ( ) No

What is the maximum number of years of CTSA/KL2 grant support that KL2 scholars can receive in your current program?

In the past, what was the typical duration of support for your KL2 graduates? (number in years)

In the past, what was the maximal duration of support for your KL2 graduates? (number in years)

What options do you currently **have, using institutional funds, to extend scholars' support (at funding levels equivalent to the KL2) for a longer duration than provided by CTSA/ KL2 grant funds?**

- Institutional support prior to KL2 appointment
- Institutional support after KL2 appointment
- No institutional scholar support before or after duration of KL2

Do you have any other parallel internal or extramurally funded KL2 equivalent career development programs?

- Yes
- No

Which training opportunities (regardless of sponsorship) are offered currently to your KL2 scholars? (check all that apply):

- Research in Progress
- Grant writing
- Leadership training
- Mentorship training
- Scientific writing
- Team Science
- Entrepreneurship
- Off-Site experiences (eg. externships, mini-sabbaticals)
- Other

Can scholars with individual K awards (or equivalent), access KL2 programming (as described above)?

- Yes
- No

How, if at all, do KL2 graduates commonly contribute to your CTSA? (check all that apply)

- Acting as mentors
- Facilitating K writing groups
- Review pilot award applications
- Other (please specify)

Are faculty compensated in any way to mentor KL2 scholars?

- Yes
- No

What is the minimum size of the KL2 mentor team?

What is the typical frequency that the scholar meets with any member of the mentoring team?

What is the typical frequency the scholar meets with the

entire mentoring team?

What is the typical frequency the scholar, along with any mentors, meets with KL2 leadership?

Is a mentor training program currently required for the mentors of KL2 scholars?

Yes
No

Is a formal Individual Development Plan (IDP) currently required for each KL2 scholar?

Yes
No

Do you recommend a specific IDP template?

Yes
No

Scholar Factors (appointed to date upon enrollment in KL2 program)

I was unable to collect the data on this table (check all that apply)

Unable to collect data, does not exist

Of all your scholars to date, approximately what percent had the following Doctoral degrees upon enrollment in the KL2 Program? (percentages should total 100%)

Attachment: Scholars who enrolled in KL2 programs with the following degrees 01_17_2017.docx (0.01 MB)

Please complete and upload the table describing the Doctoral degrees held by all of your CTSA KL2 scholars appointed to date here.

Upload document

Primary employment and rank at the time of KL2 appointment

Approximately what percent of KL2 scholars appointed to date entered with the following primary employment and rank types.

Attachment: Primary Appt Table_v01_17_2017.docx (0.01 MB)

Please complete and upload the table on KL2 primary appointments here.

Upload document

KL2 scholar characteristics

Please complete and upload the table on KL2 applicants and awardees by gender and URM status in the next field.

Attachment: KL2 Applicants and Awardees by Gender and URM Status.docx (0.01 MB)

Please complete and upload the table on gender and URM status here

Upload document

Of all your KL2 scholars to date, approximately what percent enrolled in the following during their KL2 period of support?

MS program

PhD program

Certificate program

Individual courses

No formal coursework completed

Of all of your scholars to date, approximately what percent completed a degree or certificate in clinical /translational research (noted above) prior to KL2 training?

Either a degree or certificate prior to KL2 enrollment

Of your KL2 scholars to date, approximately what percentage shortened their KL2 support to less than two years by immediately transitioning to:

Individual career development award (eg. K08, K23, foundation)

R01 or equivalent

Following at least two years of KL2 support, approximately what percentage of KL2 scholars transitioned within one year of KL2 completion to:

Individual career development award (eg. K08, K23, foundation)

R01 or equivalent

Please complete and upload the table describing the KL2 awardees extramural research funding received by your scholars during and immediately after their period of KL2 support in the next field.

Attachment: KL2 Awardees Extramural Research Funding During and After Their Period of KL2 Support.docx (0.02 MB)

Please complete and upload the table on KL2 extramural funding here.

Upload document

Career Activities after KL2 completion (time period- up to 3 years after completion of both grant and any linked institutional support)

Please complete the following table on career activities within the first three years of KL2 program completion and upload the table in the next field

Attachment: Career Trajectory table_01_17_2017.docx (0.02 MB)

Please complete and upload the table on KL2 career activities here.

Upload document

Please indicate which individuals were involved in gathering the data for this survey (check all that apply):

- [ ] KL2 Coordinator
- [ ] KL2 Director
- [ ] KL2 PI
- [ ] CTSA PI
- [ ] Evaluator
- [ ] Administrative Assistant
- [ ] Student Hourly (or other part-time assistant)
- [ ] Other (please indicate)

Please estimate the number of person hours needed to gather the data for this survey.

Please feel free to include here any comments about your experience completing this survey (e.g., clarity of particular survey questions, ease of gathering the data, potential value of the aggregate data for your hub, special circumstances we should know about your responses)

Submit

Save & Return Later

SURVEY DATA TABLES

CAREER TRAJECTORY TABLE

INSTRUCTIONS: In these tables, please count "KL2 completion" as the time each scholar completed all KL2 related training including that supported by the KL2 and any linked institutional support. Please report data separately for those scholars who are 0-3 years post KL2 completion and those > 3 years post completion. Estimates are acceptable. We do not anticipate that you will attempt to get scholar-by-scholar information specifically beyond that which you have already tracked or obtained.

SUMMARY

Total # of KL2 graduates to date	
# of KL2 graduates who completed ≤ 3 years ago	
# of KL2 graduates who completed > 3 years ago	

CURRENT LOCATION/EMPLOYMENT OF YOUR GRADUATES (estimates ok)

CURRENT LOCATION/EMPLOYMENT	Number of KL2s ≤ 3 years following completion	Number of KL2s > 3 years following completion
Currently at your CTSA institution		
Moved to a different academic institution		
Moved to non-academic employment (Government/non-Profit)		
Moved to non-academic employment (Private sector/industry)		
Moved to non-academic employment (Hospital /other)		
Other: unknown/ no longer in workforce / lost to follow-up		

TRANSLATIONAL RESEARCH EFFORT (estimates ok)

Effort devoted to Clinical & Translational Research (C&TR)	Number of KL2s ≤ 3 years following completion	Number of KL2s > 3 years following completion
No effort in C&TR		
< 30% effort In C&TR		
> 30% effort in C&TR		
Engaged in C&TR (% effort unknown)		

KL2 Applicants and Awardees by Gender and URM Status (Life of Program)		
**Gender**		
	**# Applicants**	**# Accepted**
Male		
Female		
**URM Status***		
	**# Applicants**	**# Accepted**
URM		
Non-URM		
Unknown		
*__URM__=African-American/Black; Hispanic/Latino; American Indian or Alaskan Native; Native Hawaiian or Other Pacific Islander; Two or more races (if one of those is underrepresented); and Persons with Disabilities as defined by the ADA<br>**Non-URM**=White; Asian		

KL2 Awardees Extramural Research Funding During and After Their Period of KL2 Support						
	**Approximate % of KL2 scholars to date that were awarded extramural funding** <u>during</u> **the KL2**		**Approximate % of KL2 scholars to date that were awarded extramural funding** <u>within a year after</u> **the KL2**		**Approximate % of KL2 scholars to date that were awarded extramural funding** <u>within 5 years</u> **of the KL2**	
**Source of Extramural Funding (total should add up to 100%)**	**As PI**	**As Co-I**	**As PI**	**As Co-I**	**As PI**	**As Co-I**
NIH award						
Other Federal Award (Ex. VA, AHRQ, CDC, NSF)						
Foundation/Professional society grant (Ex. AHA, ACS, RWJ, Doris Duke)						
Commercial Sponsored Research						
No extramural funding						
No information available	----	----				
**Specific Funding Mechanisms**	**As PI**	**As Co-I**	**As PI**	**As Co-I**	**As PI**	**As Co-I**
Major Research Project Grants (Ex. R01, PPG, VA Merit or equivalent)						
Other Research Project Grants (Ex. R03, R21 or equivalent)						
Individual career development awards, federal or foundation						
Cooperative Agreements and Contracts						

PRIMARY APPOINTMENT TABLE

Estimated Percent of total KL2 Scholars with the following Primary Appointments at the start of the KL2 program (chose only one appointment) (%)	
Scientist\ Researcher	%
Academic \ faculty title (Assistant Professor and beyond, any track or type)	%
Lecturer \ Instructor or "pre-faculty title"	%
Hospital title (with or without academic appointment)	%
Other	%

Please estimate the percent of your scholars to date who had the following training at the start of the KL2 program

Estimated Percent of scholars to date with the following training at the start of the KL2 program (SHOULD TOTAL 100%)	
**CLINICAL** training alone (e.g., MD, DVM, DDS, PharmD, etc)	%
**RESEARCH** training alone (e.g., non-clinical PhDs)	%
**BOTH** clinical and research training (e.g., MD/PhD, MD/MS, MD/MPH)	%
